# Cost-Effectiveness of Lung Cancer Screening with Low-Dose Computed Tomography: Comparing Hungarian Screening Protocols with the US NLST

**DOI:** 10.3390/cancers16172933

**Published:** 2024-08-23

**Authors:** Tanya Rajabi, László Szilberhorn, Dávid Győrbíró, Manna Tatár, Zoltán Vokó, Balázs Nagy

**Affiliations:** 1 University of Rochester School of Medicine and Dentistry, Rochester, NY 14642, USA; tanya_rajabi@urmc.rochester.edu; 2 Center for Health Technology Assessment, Semmelweis University, 1091 Budapest, Hungary; toth.manna@phd.semmelweis.hu (M.T.); voko.zoltan@semmelweis.hu (Z.V.); 3 Syreon Research Institute, 1142 Budapest, Hungary

**Keywords:** low-dose computed tomography, lung cancer, screening, cost-effectiveness, public health, decision modeling

## Abstract

**Simple Summary:**

Independent evaluations have demonstrated the cost-effectiveness of lung cancer screening with LDCT, yet few studies have directly compared the cost-effectiveness of different screening protocols. The aim of our study was to compare the cost-effectiveness of Hungarian screening protocols (based on the NELSON trial) and the US NLST applied in the same healthcare context. This was carried out using variations of the same decision-analytic model. We demonstrated that a less conservative and more rapid diagnostic strategy for screening can be cost-effective for Hungary. We further discussed the advantages and disadvantages of the NLST and NELSON LDCT screening protocols on the matters of patient uncertainty, diagnostic timelines, overdiagnosis, and target group identification. With many countries considering the introduction of national lung-cancer-screening programs, insights from our study can be used to tailor programs to individual countries’ needs and healthcare capacities.

**Abstract:**

We aimed to directly compare the cost-effectiveness of Hungarian (following the NELSON trial) and NLST screening protocols, two trials influencing lung-cancer-screening implementation internationally. A decision-analytic model analyzing the cost-effectiveness of Hungarian protocols was manipulated to reflect the protocols of the NLST, while maintaining features specific to the Hungarian healthcare setting. In the Hungarian protocol, there are three possible outcomes to the initial round of screening, positive, negative, and indeterminate, indicating an uncertain degree of suspicion for lung cancer. This protocol differs from the NLST, in which the only possible screening outcomes are positive or negative, with no indeterminate option. The NLST pathway for smokers aged 55–74 resulted in a EUR 43 increase in the total average lifetime costs compared to the Hungarian screening pathway and resulted in a lifetime gain of 0.006 QALYs. The incremental costs and QALYs yielded an ICER of 7875 EUR/QALY. Our results demonstrate that assigning any suspicious LDCT screen as a positive result (NLST protocol) rather than indeterminate (Hungarian protocol) can reduce patient uncertainty and yield a slight QALY gain that is worth the additional use of resources according to Hungary’s willingness-to-pay threshold. A stratified analysis by age was also conducted, revealing decreasing cost-effectiveness when screening older cohorts. Our study provides insight into the cost-effectiveness, advantages, and disadvantages of various LDCT screening protocols for lung cancer and can assist other countries as they implement their screening programs.

## 1. Introduction

Lung cancer is the leading cause of cancer deaths globally [1], with Hungary being in the mid-league among European countries, at an overall incidence rate of 52.4 per 100,000 person-years [2,3]. Due to late-appearing symptoms, only 16% of lung cancers are diagnosed early, while most cases are diagnosed at a metastatic stage, in which treatment options are ineffective and the five-year survival rate is 8% [4,5].

In the past two decades, low-dose computed tomography (LDCT) has gained attention internationally as a tool for screening and diagnosing lung cancer at curable stages. The US National Lung Screening Trial (NLST) and the European NELSON trial provided evidence that screening by LDCT reduces lung cancer mortality by 20% and 25%, respectively [6,7]. In Eastern Europe, the HUNCHEST pilot trial demonstrated that LDCT screening facilitates early lung cancer diagnosis [8]. However, there has been debate on which LDCT screening protocols are the most efficient in diagnosing lung cancer and whether these protocols are sufficiently cost-effective to warrant routine screening for the millions at risk.

In Hungary, annual LDCT screening for smokers was demonstrated to be cost-effective [9]. Various independent evaluations of the NLST have also demonstrated cost-effectiveness for the United States [10,11]. However, there are inherent differences in the screening protocols currently practiced in Hungary, which are similar to those used in the NELSON trial and the screening protocols used in the NLST. Most notable is the option for an indeterminate result in Hungary for a scan potentially suspicious for lung cancer, which is confirmed as positive or negative in 3 months by a repeat LDCT scan [8]. In the NLST, all screens are deemed either positive or negative, with no option for a repeat LDCT scan [6]. Regarding the clinical diagnosis of lung cancer, Hungary and the rest of Europe diagnose cases based on the nodule volume and the volume-doubling time, while the United States assesses the nodule diameter [12]. These differences in screening procedures can have implications for diagnosis rates and timelines, resource consumption, and patient outcomes.

With many countries considering the introduction of LDCT screening for lung cancer, it is important to assess the different screening protocols practiced internationally. Despite several independent evaluations of cost-effectiveness in the United States and Europe, few studies have directly compared the cost-effectiveness of different screening policies. In this paper, we aimed to directly compare the cost-effectiveness of Hungarian and NLST screening strategies applied in the same healthcare environment.

## 2. Materials and Methods

### 2.1. Model Development

A decision-analytic model analyzing the cost-effectiveness of Hungarian lung-cancer-screening protocols, based on the NELSON protocol, was originally developed by Nagy et al. (2023) [9]. We manipulated this model to reflect the protocols of the NLST, while maintaining features specific to the Hungarian healthcare setting. This was done to estimate the lifetime costs and patient outcomes of annual LDCT screening had Hungary followed the screening protocols used by the NLST. Throughout the paper, the “Hungarian screening pathway” or the “Hungarian model” refers to the original model reflecting Hungarian protocols used in the Hungarian setting. The “US screening pathway” or the “US model” refers to the manipulated model reflecting NLST protocols used in the Hungarian setting.

**Figure 1:**
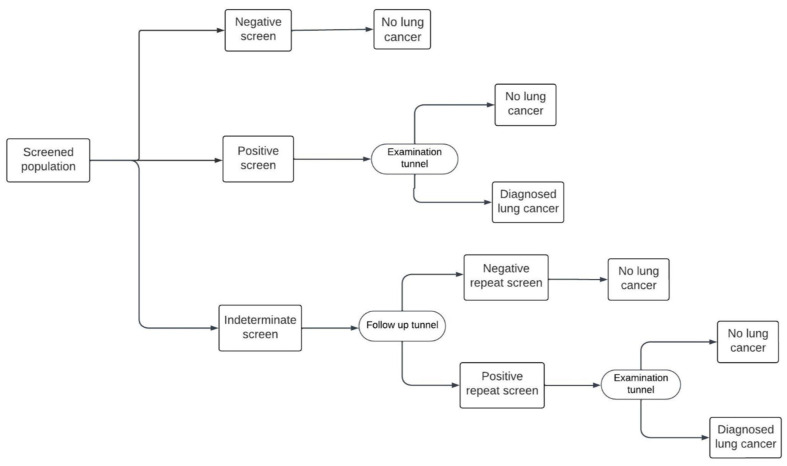
The Hungarian model screening pathway.

**Figure 2:**
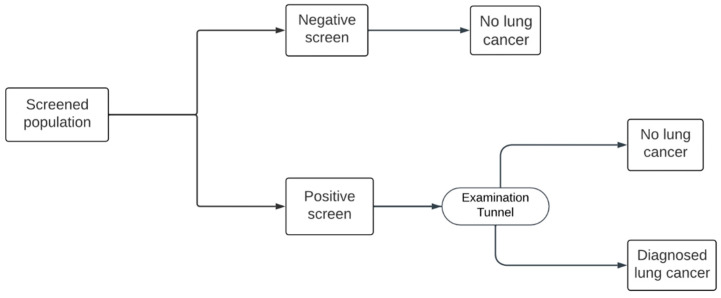
The US model screening pathway.

Both the Hungarian and US models include an initial baseline screening, followed by annual screening from age 55 up to the age of 74. In the Hungarian model, there are three possible outcomes to the initial round of screening, positive, negative, and indeterminate, indicating an uncertain degree of suspicion for lung cancer (Figure 1). This protocol differs from the US model, in which the only possible screening outcomes are positive and negative, with no indeterminate option (Figure 2). In both models, patients with a positive result enter an “Examination tunnel”, entailing more intensive examinations for lung cancer diagnosis, such as a chest or abdominal CT scan, bronchoscopy, or biopsy. Meanwhile, patients with an indeterminate result in the Hungarian model are subject to a follow-up LDCT screen in 3 months; next, they are assigned a final negative result or a positive result and subsequently enter the “Examination tunnel”. The cycle length of both models is 1 month. Patient pathways after diagnosis and subsequent (beyond year 1) screening years are simulated in the decision-analytic model, as explained in detail by Nagy et al. [9].

### 2.2. Effectiveness Data and Model Inputs

Effectiveness data for screening in the Hungarian model were based on data from the HUNCHEST study [8], the NELSON trial [13,14,15], and Hungarian incidence and prevalence data [16,17]. To develop the US model, data from the NLST were derived from the National Institute of Health’s Cancer Data Access System [18]. Effectiveness data for screening in the US model were calculated from the NLST’s baseline and year 1 LDCT screening results, which included 26,309 patients screened from August 2002 through April 2004. These data were used as model inputs for patient pathways, as can be seen in Table 1 and Table 2. The NLST cohort included either current or former smokers (who had quit within the past 15 years) aged 55–74 years with a 30+ pack-year history. This is comparable to the Hungarian model’s cohort of current smokers aged 55–74 years with a 25+ pack-year history.

Given the absence of an indeterminate status in the NLST, the rate of indeterminate screens was 0% in the US model; hence, all screens were either positive or negative (Table 1 and Table 2). This contrasts with the Hungarian model, in which an indeterminate result was assigned for 14.35% and 6.58% of baseline and year 1 screens, respectively. A greater proportion of patients were therefore screened positive in the US model (27.33%) compared to the Hungarian model (3.33%).

The rates of diagnostic examinations for the “Examination tunnel” in the US model are derived from the procedure rates conducted in the NLST’s baseline year (Table 3). The examination rates in the Hungarian model are based on the published literature and expert opinion [19]. The difference in examination patterns is a reflection of the different screening protocols, as well as each country’s medical system. Healthcare costs associated with LDCT screening and diagnosis are based on Hungarian national health insurance data and were unchanged in the Hungarian and US models [20,21,22]. Treatment data for diagnosed cases were unchanged between models to maintain Hungarian medical practices.

### 2.3. Utility and ICER Calculation

The models estimate patients’ utility combined with their life trajectory to yield quality-adjusted life years (QALYs). In both models, these were calculated using Hungarian EuroQol 5D index values and international data sources that were adjusted to age- and gender-specific utility tariffs [23]. A 0.03 utility decrement was associated with the uncertainty of waiting for a repeat LDCT in indeterminate cases in the Hungarian model [24,25].

The models calculate costs and the incremental cost–effectiveness ratio (ICER). All costs and benefits were discounted at a yearly rate of 3.7% [26]. Results are presented in euros based on the average conversion rate of 2023 for Hungarian Forints to euros (EUR 1 = HUF 381.74) [27]. The willingness-to-pay threshold for this particular indication according to the latest Hungarian guideline is EUR 26,761 (1.5 × GDP/capita) [26].

### 2.4. Deterministic Sensitivity Analysis

One-way deterministic sensitivity analysis was used to test parameter uncertainty. Parameters were changed one-by-one by +/−10% of the default value: a tornado diagram presented the most sensitive parameters (Figure 1 and 2). Additionally, a stratified analysis of age was carried out, accounting for differences in lung-cancer-screening effectiveness among different age bands in the NLST (Table 4).

### 2.5. Overdiagnosis

Both models track screen-detected and clinically detected cases separately. As such, we could estimate overdiagnosis (defined as the probability that a screen-detected cancer would not have been diagnosed clinically in the patient’s lifetime without screening) for both US and Hungarian pathways using the method proposed by Patz et al. [28]: number of excess lung cancer cases in the LDCT arm compared to the no-screening arm/total number of screen-detected lung cancer cases in the LDCT arm.

## 3. Results

### 3.1. Diagnosis Rate

Our results showed that if smokers aged 55–74 years were screened annually under the Hungarian pathway, the proportion of diagnosed lung cancer cases would be 3.53% after five years (Table 5). If smokers were screened annually under the US pathway, the proportion of diagnosed cases would be slightly greater, at 3.61%, which yields a 2.21% relative increase in the proportion of detected cases.

### 3.2. ICER

The average lifetime treatment cost (the cost of diagnostic examinations and subsequent lung cancer treatment) of the population of smokers aged 55–74 years screened annually under the Hungarian pathway was previously calculated to be EUR 2317, and the average lifetime screening cost (the cost of initial and repeat LDCT screening) was calculated to be EUR 226 [9]. Annual screening for the same population under the US screening pathway resulted in an increased average treatment cost of EUR 2375 and a decreased average screening cost of EUR 211. Consequently, the US pathway resulted in a EUR 43 increase in total average lifetime costs (from EUR 2543 to EUR 2586). The US screening pathway for smokers aged 55–74, compared to the Hungarian screening pathway, resulted in a lifetime gain of 0.006 QALYs. The incremental costs and QALY yielded an ICER of 7875 EUR/QALY (Table 6).

### 3.3. Overdiagnosis

The probability of overdiagnosis under the US pathway was found to be 8.39% in comparison to the Hungarian pathway’s overdiagnosis rate of 7.93%, which reflects a 5.80% increase in overdiagnosis rates.

### 3.4. Deterministic Sensitivity Analysis

**Figure 3:**
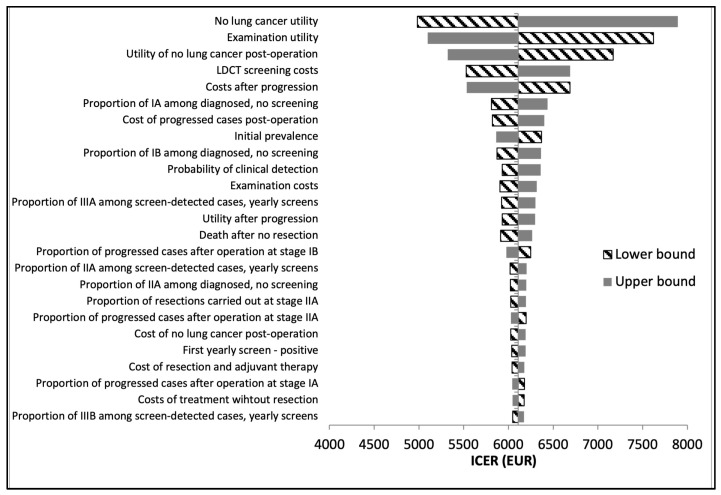
US model: deterministic sensitivity analysis tornado diagram. Note: ICERs reflect the comparison of the US annual screening pathway vs. no screening.

**Figure 4:**
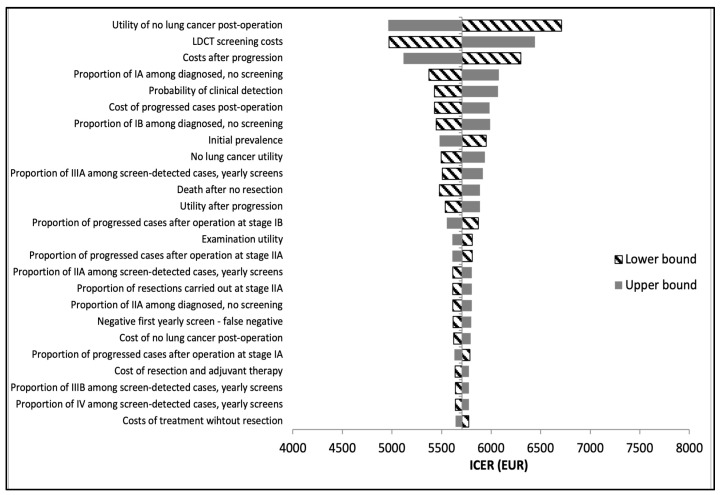
Hungarian model: deterministic sensitivity analysis tornado diagram. Note: ICERs reflect the comparison of the Hungarian annual screening pathway vs. no screening.

One-way deterministic sensitivity analysis demonstrated that when compared to no lung cancer screening at all, the most influential parameters in the US model are the utility value of not having lung cancer, the utility of undergoing diagnostic examinations, the utility of no lung cancer post-operation, LDCT screening costs, and treatment costs after the progression of lung cancer (Figure 3). The Hungarian model demonstrated the same most influential parameters when compared to no screening, apart from the utility of not having lung cancer and the utility of diagnostic examinations, which were the 9th- and 14th-most influential, respectively (Figure 4). Rather, the probability of clinical detection was in the top influential parameters in the Hungarian model only.

### 3.5. Stratified Analysis by Age

Stratified analysis of age bands (Table 7) demonstrated that when selectively screening the population of ages 55–64, there was a QALY gain of 0.80 and a cost decrease of EUR 173, yielding cost savings when compared to the base case cohort of 55–74 years. Selectively screening the population of ages 55–69 also demonstrated a QALY gain and cost savings but to a lesser extent than the 55–64 age group. We saw a switch in the age bands 60–74 and 65–74, where there were fewer QALYs and more costs compared to the base case. Screening a younger subset of the population was deemed more efficient than screening the 55–74-year-old population.

## 4. Discussion

In this study, we explored the economic impact of applying the US (NLST) protocol for lung cancer screening in the Hungarian healthcare setting compared to the currently applied European (NELSON) protocol. A fundamental difference between the two programs is the way uncertain cases are handled. The NELSON protocol assigns uncertain cases to an indeterminate health state and repeats LDCT screening before the case undergoes a final diagnosis. In contrast, the NLST protocol directs uncertain cases straight through the standard process of lung cancer diagnosis (termed “Examination tunnel” in our analysis).

Our results demonstrate that assigning any suspicious LDCT screen as a positive result (US model) rather than indeterminate (Hungarian model) can yield a slight QALY gain that is worth the additional use of resources according to Hungary’s willingness-to-pay threshold. In other words, the Hungarian health system may benefit by forgoing a repeat LDCT screen at 3 months and instead jumping to a more advanced diagnostic test, such as a CT scan or a bronchoscopy. However, it is important to note that compared to no screening, both the NLST and the NELSON screening protocols are deemed cost-effective in the Hungarian setting.

Applicable to the Hungarian model only, patients with an indeterminate screen have a utility decrement of 0.03 while waiting for their repeat LDCT. This follows the logic that the state of uncertainty associated with waiting an additional 3 months for final screening results is less desirable than receiving an initial, definitive result [24,29]. Patients in the US model do not experience this utility loss, as they receive a confirmed positive or negative result in the initial screening round. Therefore, as there is no follow-up LDCT screen in the US model, the elimination of uncertainty largely explains the QALY gain demonstrated in favor of the US model.

National screening programs have been implemented due to the importance of early detection in treating and surviving lung cancer [30,31]. It is assumed that in our study’s US model, lung cancer can be diagnosed earlier, as suspicious or uncertain cases immediately undergo diagnostic examinations (abdominal or chest CT, bronchoscopy, or biopsy). In terms of diagnostic timelines, the US model has an approximately 3-month advantage over the Hungarian model due to not requiring a repeat LDCT screen for indeterminate cases. Treatment can therefore begin earlier in the US case, potentially increasing the chances of survival [32]. More patients in the US model are thus captured in the “Examination tunnel” and undergo advanced diagnostic examinations. Our deterministic sensitivity analysis validates this (Figure 3), as the utility of advanced diagnostic examinations was among the top influential parameters in the US model only. This translates to increased spending necessitated by the NLST protocol, as evidenced by the increased average treatment cost in the US model. However, our results demonstrate that the slight overall increase in the total average lifetime costs is worth it, i.e., the cost of gaining one QALY is below Hungary’s societal willingness-to-pay threshold. An analysis by Nagy et al. in 2021 demonstrated that the introduction of lung cancer screening in Hungary would cost an estimated average 6.8 million EUR/year but could result in cost savings in the long run [33]. Similarly, evaluating the budget impact of adopting NLST protocols in Hungary would be informative in comparing the long-term macroeconomic effects of screening. While we anticipate that yearly costs may initially be higher under NLST protocols, greater savings may be achieved over time due to the earlier delivery of treatment.

It is also important to consider minimizing resource overuse and unnecessary patient radiation, as overdiagnosis and high false-positive rates are often a point of criticism in routine LDCT screening [34,35]. Overdiagnosis rates in the US model increased in comparison to the Hungarian model, which is expected, given the higher positive rate among first screens. Notable in the input tables (Table 1 and Table 2) is also the greater rate of false-positive cases in the US model compared to the Hungarian model (96.25% vs. 51.02% in the baseline year and 97.57% vs. 52.67% in year 1), due to the lack of an indeterminate screening result in the US model. In line with these findings, our deterministic sensitivity analysis showed that the probability of clinical detection is a top parameter for only the Hungarian model. This confirms that clinical detection, i.e., cases caught without screening, plays a larger role in lung cancer diagnosis in the Hungarian protocol than in the US model, influenced by the lower positive rate in the Hungarian model. Identification of appropriate target groups for screening is an important step in limiting overdiagnosis and false positives.

Hungary and the rest of Europe examine the nodule volume and volume-doubling time for diagnosing suspicious nodules, and studies have shown that these volume-based methods have higher predictive values and reduced false-positive rates compared to diameter-based methods [12,36]. Throughout the NLST, however, a diameter-based analysis for nodule diagnosis was used, and the United States continues to use this method today. Therefore, the method of diagnosis may also be partially responsible for the high false-positive rates observed in the NLST. Ongoing studies are evaluating the impact of the United States transitioning to volume-based diagnosis [37,38]. If false-positive rates can be reduced by changing to the volume-based diagnostic method, the cost-effectiveness of LDCT screening under US screening protocols may be even greater than what we demonstrate in this study.

Our model accounted for initial incidence and mortality data specific to various age groups, allowing us to compare their LDCT screening outcomes and costs. Stratified analysis showed that a more targeted approach to screening based on age decreases costs incurred and increases the QALY gain. Even if false-positive rates are lower for the older age bands (mainly due to higher incidence of lung cancer), detection among this population leads to less gains as their lifespans are shorter and they receive less benefit from treatment [39]. These results are in line with the published literature suggesting that the value of routine LDCT screening may begin decreasing over 65 years of age [40], and here, we demonstrated the decreased economic value, too.

While we were able to stratify based on age, incidence, and mortality data for other factors known to influence lung cancer outcomes, including COPD, the status as a current versus former smoker and the number of pack-years smoked [41,42] were not included in the model, and thus, we were unable to stratify based on these factors. A future version of the model can account for these risk factors to comprehensively evaluate cost-effectiveness for various cohorts [43]. Incorporating regional differences in the lung cancer incidence in Hungary can also be informative in future models, as was evidenced in a study by Gálffy et al. [44]. Furthermore, risk prediction models for LDCT screening can allow for personalization to individual needs, as is being assessed in the 4-IN-THE-LONG-RUN trial [45].

Lastly, the NLST was conducted from 2002 to 2004 and set the framework for a more robust national screening program in the United States. In 2014, the Lung CT Screening and Reporting Data System (Lung-RADS) was implemented to guide physicians and standardize the lung-cancer-screening process in the United States [46]. This includes recommendations for follow-up screening tailored to the nodule size and growth over time. Therefore, we expect to see an improvement in false-positive and overdiagnosis rates today in the United States. However, it is important to reflect on the NLST itself, as the trial continues to be a guiding force internationally for the many countries just now implementing their own trials and screening programs.

It is important to emphasize that our results reflect the healthcare setting of Hungary, with Hungarian healthcare costs and utilities applicable to the Hungarian population. While we demonstrated that a less conservative and more rapid diagnostic strategy to screening can be cost-effective for Hungary, this may not be the case for all countries. Individual countries should assess what works best within the capacities of their own healthcare infrastructures and budgets, as well as their populations’ needs. Nevertheless, our study provides insight into the cost-effectiveness, advantages, and disadvantages of two influential screening trials and can be used as a resource for other countries beginning their national screening programs.

## 5. Conclusions

Using an appropriate lung-cancer-screening strategy is imperative to maximizing clinical efficacy and cost-effectiveness. Our results demonstrate the cost-effectiveness of accelerating screening timelines, while minimizing patient uncertainty in Hungary. However, balancing these benefits with the drawbacks of overdiagnosis and resource overuse remains a challenge. As screening with low-dose computed tomography continues to demonstrate reductions in lung cancer mortality, focus should shift toward comparing the clinical efficacy and economic impact of various methods in individual healthcare settings.

## Figures and Tables

**Table 1:** Baseline screening effectiveness data and model inputs: Hungarian model vs. US model.

Input Name	Hungarian Model N = 1860	US Model N = 26,309
First baseline screen—positive	62 (3.33%)	7191 (27.33%)
First baseline screen—negative	1531 (82.31%)	19,118 (72.67%)
First baseline screen—indeterminate	267 (14.35%)	0 (0.00%) ^1^
Negative first baseline screen—true negative	591 (99.83%) ^2^	19,100 (99.91%)
Negative first baseline screen—false negative	1 (0.17%)	18 (0.09%)
Positive first baseline screen—true positive	24 (48.98%)	270 (3.75%)
Positive first baseline screen—false positive	25 (51.02%)	6921 (96.25%)
Indeterminant first baseline screen—positive repeat ^3^	13 (12.50%)	0 (0.00%) ^1^

^1^ No indeterminate result in the US model; ^2^ 939 patients lost to follow-up; ^3^ follow-up LDCT screen after 3 months. Source: Nagy et al. [9], HUNCHEST data [8], NLST database [18].

**Table 2:** Year 1 screening effectiveness data and model inputs: Hungarian model vs. US model.

Input Name	Hungarian Model N = 682	US Model N = 24,715
First yearly screen—positive	9 (1.25%)	6904 (27.93%)
First yearly screen—negative	629 (92.17%)	17,811 (72.07%)
First yearly screen—indeterminate	44 (6.58%)	0 (0.00%) ^1^
Negative first yearly screen—true negative	68 (99.90%) ^2^	17,801 (99.94%)
Negative first yearly screen—false negative	1 (0.10%)	10 (0.06%)
Positive first yearly screen—true positive	4 (47.33%)	168 (2.43%)
Positive first yearly screen—false positive	5 (52.67%)	6736 (97.57%)
Indeterminant first yearly screen—positive repeat ^3^	2 (8.33%)	0 (0.00%) ^1^

^1^ No indeterminate result in the US model; ^2^ 560 patients lost to follow-up; ^3^ follow-up LDCT screen after 3 months. Source: Nagy et al. [9], HUNCHEST data [8], NLST database [18].

**Table 3:** Proportion of positive screens undergoing various diagnostic examinations (“Examination tunnel”) and their associated costs.

Examination	Hungarian Model	US Model	Unit Cost (EUR)
Initial LDCT scan	100%	100%	52.39
Abdominal CT	39%	3%	83.43
Chest CT	52%	73%	83.43
Bronchoscopy	64%	4%	28.87
CT-guided thoracic biopsy	27%	2%	77.90

Source: Nagy et al. [9], NLST database [18].

**Table 4:** Baseline and year 1 screening effectiveness data stratified by age bands: US model.

	Baseline		Year 1	
Age Band	Positive Rate	False-Positive Rate	Positive Rate	False-Positive Rate
55–74 (reference)	27.33%	96.25%	27.94%	97.57%
55–64	25.62%	96.95%	26.25%	98.01%
55–69	26.68%	96.61%	27.39%	97.72%
60–74	29.62%	95.63%	30.31%	96.92%
65–74	32.05%	94.70%	32.63%	96.57%

Source: NLST database [18].

**Table 5:** Proportion of lung cancer cases diagnosed after years 1, 3, and 5 of annual screening.

Model	Year 1	Year 3	Year 5
Hungarian	1.64%	2.32%	3.53%
US	1.71%	2.38%	3.61%

**Table 6:** Base case results of the cost–effectiveness model.

Model	Treatment Cost (EUR) *	Screening Cost (EUR) **	Total Cost (EUR)	QALY	ICER (EUR/QALY)
Hungarian	2317	226	2543	6.996	(Reference)
US	2375	211	2586	7.002	7875

* Treatment cost includes the cost of diagnostic examinations (in the “Examination tunnel”) and subsequent treatment costs; ** screening cost includes the cost of the initial LDCT scan and the repeat LDCT scan for indeterminate cases (in the Hungarian model).

**Table 7:** Stratified analysis of age bands in the US model: results compared to the 55–74 age band.

Age Band	QALY	Δ QALY	Cost (EUR)	Δ Cost (EUR)	ICER (EUR/QALY)
55–74 (reference)	7.00	(Reference)	2586	(Reference)	(Reference)
55–64	7.80	0.80	2413	−173	−215 * (dominates)
55–69	7.48	0.48	2523	−63	−130 * (dominates)
60–74	6.10	−0.90	2729	143	−159 ** (dominated)
65–74	5.39	−1.61	2736	150	−93 ** (dominated)

* Analyzed age group dominates over the reference group (cheaper and more beneficial); ** analyzed age group is dominated by the reference group (more expensive and less beneficial).

## Data Availability

Data from the National Lung Screening Trial can be requested from the National Institute of Health Cancer Access System at https://cdas.cancer.gov/nlst/ (accessed on 21 May 2024).
